# Precision in Setting Cancer Prevention Priorities: Synthesis of Data, Literature, and Expert Opinion

**DOI:** 10.3389/fpubh.2017.00125

**Published:** 2017-06-06

**Authors:** Jennifer Girschik, Laura Jean Miller, Tony Addiscott, Mike Daube, Paul Katris, David Ransom, Terry Slevin, Tim Threlfall, Tarun Stephen Weeramanthri

**Affiliations:** ^1^Public Health Division, Department of Health Western Australia, Perth, WA, Australia; ^2^Health Consumers Council, Perth, WA, Australia; ^3^Faculty of Health Sciences, Curtin University, Perth, WA, Australia; ^4^Western Australian Clinical Oncology Group, Perth, WA, Australia; ^5^Cancer and Palliative Care Network, Department of Health Western Australia, Perth, WA, Australia; ^6^Cancer Council Western Australia, Perth, WA, Australia; ^7^Western Australian Cancer Registry, Department of Health Western Australia, Perth, WA, Australia

**Keywords:** cancer prevention, cancer control, preventability, prioritization, policy, public health

## Abstract

Cancer will continue to be a leading cause of ill health and death unless we can capitalize on the potential for 30–40% of these cancers to be prevented. In this light, cancer prevention represents an enormous opportunity for public health, potentially saving much of the pain, anguish, and cost associated with treating cancer. However, there is a challenge for governments, and the wider community, in prioritizing cancer prevention activities, especially given increasing financial constraints. This paper describes a method for identifying cancer prevention priorities. This method synthesizes detailed cancer statistics, expert opinion, and the published literature for the priority setting process. The process contains four steps: assessing the impact of cancer types; identifying cancers with the greatest impact; considering opportunities for prevention; and combining information on impact and preventability. The strength of our approach is that it is straightforward, transparent and reproducible for other settings. Applying this method in Western Australia produced a priority list of seven adult cancers which were identified as having not only the biggest impact on the community but also the best opportunities for prevention. Work conducted in an additional project phase went on to present data on these priority cancers to a public consultation and develop an agenda for action in cancer prevention.

## Introduction

Cancer is a major cause of ill health and death in Western Australia (WA). Almost 12,000 Western Australians are diagnosed with cancer and around 4,000 lose their lives to the disease every year (1). In addition, approximately 85,000 non-melanoma skin cancers are treated each year in WA (2). Consequently, cancer is also a major source of government expenditure. The most recent national report estimates that total health system expenditure on cancer (excluding population screening programs) was $4.5 billion in 2009 (3). Recent studies have estimated that 30–40% of cancers could be prevented (4, 5), which, if achieved, would save much of the pain, anguish, and cost associated with treating cancer.

The Western Australian State Health Department is responsible for establishing policies for cancer prevention in the state. As in most government departments, this responsibility is being enacted in an environment that demands a robust evidence base along with collaboration, transparency, and accountability in decision-making, all within increasing fiscal constraints. This can be a difficult undertaking in any area, but can be uniquely complicated for cancer prevention.

Specifically, the evidence base for prioritizing between cancer prevention targets is not always obvious. Determining the relative impact of the different types of cancer would, in theory, highlight the areas where resources are most needed. However, cancer impact can be measured across a range of domains including incidence, morbidity, duration, mortality, years of potential life lost and cost, and the choice of domain can impact significantly on the relative priority of different cancers. In addition, impact alone is not sufficient to drive public health expenditure if scientifically proven prevention strategies are not available (6). Additionally, cancer prevention involves a range of organizations, services, and expertise, all of which have their own perspectives, priorities, and funding constraints, and may not easily sit together. Moreover, public preferences and community values play a role in the allocation of public resources, for both practical reasons around policy effectiveness and ethical reasons around civic participation and democratic legitimacy (7). In some instances, public opinion and behavior can be at odds with the available evidence on cancer prevention (8–11). For example, screening programs regularly report participation rates well below the target rate, despite the programs being publicly funded and having well-documented population benefits (12–14). Above all, there is significant pressure to ensure the best return for investment.

In this context, how do policy makers appropriately appraise and balance these complex and sometimes competing demands to ensure policy development that will maximize public good and ensure the best return for the limited cancer prevention dollar?

This was a challenge faced in the Western Australian Department of Health (WA DoH); the solution was to develop a method for, and undertake a process of, priority setting through a project called *Priorities and Preferences for Cancer Control in Western Australia*. The project was conducted by the Public Health Division under the auspices of the Chief Health Officer. The ultimate aim of the project was to develop a list of cancer prevention priority areas in WA informed by evidence, expert opinion, and public priorities and preferences. This list could then serve as the basis for more technical discussions around appropriate prevention/health promotion strategies, including issues of cost-effectiveness, delivery, and evaluation. To achieve this aim, the project was conducted in two phases. The first phase identified a priority list of cancers with both the biggest impact on the WA community and the best opportunities for prevention. The second phase involved using evidence of the impact and preventability of our priority cancers to engage the public in a discussion around their preferences for cancer control in the state.

This paper describes the first phase of the project, the process of identifying and prioritizing cancers. It is hoped that our perspective paper will inform others who may also be challenged by the process of priority setting in cancer prevention.

## Establishing an Expert Advisory Group

The initial step in this project was the establishment of an expert advisory group to provide oversight and review of the analysis and interpretation undertaken by the project staff. The expert advisory group consisted of eight local cancer experts representing a range of relevant disciplines, including prevention, health promotion, clinical oncology, cancer registration, epidemiology, consumer advocacy, and policy development. The advisory group met regularly and reviewed all aspects of the project from analysis through community consultation and finally to dissemination of the project findings.

## Accessing and Analyzing Data

The second task involved intensive data review and analysis in an attempt to describe the impact of cancer on the WA community. WA, like most Australian states, has a dedicated population-based cancer registry whose legislative mandate is to collect and collate information on diagnoses and deaths from cancer in WA (1). De-identified data provided by the WA Cancer Registry (WACR) was central to much of the analysis describing the impact of cancer in WA. Approval for the project and access to the de-identified data were granted by the WACR Data Custodian, in accordance with our institutional ethics committee terms of reference (15).

Statistics for 55 different cancer types recorded in the WACR were generated by the project team including age-standardized incidence and mortality rates, person years of life lost, and hospital length of stay for 2012 (data extracted December 2013). In addition, relative 5-year survival was calculated for three 5-year time periods: 1986–1990, 1996–2000, and 2006–2010. These statistics were calculated for males and females combined and separately. In addition, trends over time were calculated for incidence, mortality, and survival.

Data from outside the scope of the WACR was also sought, including Burden of Disease and costing information. Disability-adjusted life years for 2012 for WA were available as projections from the 2006 WA Burden of Disease study (16). Burden of Disease data were extracted for men and women combined and separately. Estimated lifetime treatment cost per case and total health system cost were also extracted from a national report produced by the Australian Institute for Health and Welfare for males and females combined (17). However, there were limitations to both Burden of Disease and cost data as information was not available for all the cancer types recorded in WACR and some classifications of cancer types were different (17, 18).

International data were also sought to place the WA results in a global context. For this purpose, data from the International Agency for Research on Cancer project Globocan 2012 was used, as it is the global source with the most reliable data (19). This World Health Organization initiative produces estimates with the most recent data available at the time. Using the Globocan 2012 project’s age-standardized incidence and mortality rates for Organisation for Economic Cooperation and Development countries, of which Australia is a member, the complement of the mortality to incidence ratio (MIR) was calculated. The MIR is the best available method for international comparisons of cancer survival. Again, however, Globocan did not have information available for all the cancer types recorded in the WACR (19).

While an extensive number of data analyses were conducted, they all used standard methods and therefore can easily be replicated. The more difficult phase was determining how to compare and prioritize the varied analysis to define the cancers that have the biggest effect on the community. Should we prioritize a cancer with a high number of new cases but a low death rate above or below a cancer with a low number of cases but a high death rate? Should we prioritize a cancer with a small number of cases but a high treatment cost above or below a cancer with a short hospital length of stay but a large number of cases? And who should be making this decision—an epidemiologist, a policy maker, or the community?

## Assessing Cancer Impact

As a first step in assessing the impact of individual cancers, the expert advisory group reviewed the cancer types recorded in the WACR. From this review, the classification of esophageal and stomach cancer was changed. Whereas the WACR classified esophageal and stomach cancer separately (ICD-O codes C15 and C16 respectively), expert opinion from the advisory group argued for their amalgamation on the grounds that in Western Societies they most commonly occur at the gastroesophageal junction making the classification of “esophageal” or “gastric” somewhat arbitrary. Statistics were then recalculated for esophageal and stomach cancer combined.

As the second step in assessing the impact of individual cancers, the expert advisory group reviewed the available data to decide which variables might best reflect the overall impact of each cancer on the general population. There was consensus among the advisory group that, in general, incidence and mortality were the strongest indicators of the impact of cancer on people in the community, which is consistent with the conclusions of earlier studies that had similar aims (6, 20). In addition, the incidence and mortality datasets were derived from the WACR and we were confident in the accuracy and completeness of these datasets for the full scope of cancers. Furthermore, it was felt that incidence and mortality would be the easiest to interpret for a general audience. This was important as the second phase of this project was to present the data to the general public and facilitate a discussion around setting priorities and preferences for cancer prevention in the state. Although Burden of Disease data are considered a very good tool for priority setting, in this case, the age of the source data and the reliance on projections made it less than ideal, and there was also some concern that it would not be easily understood by a general audience.

## Identifying the Cancers with the Greatest Impact

**Figure 1:**
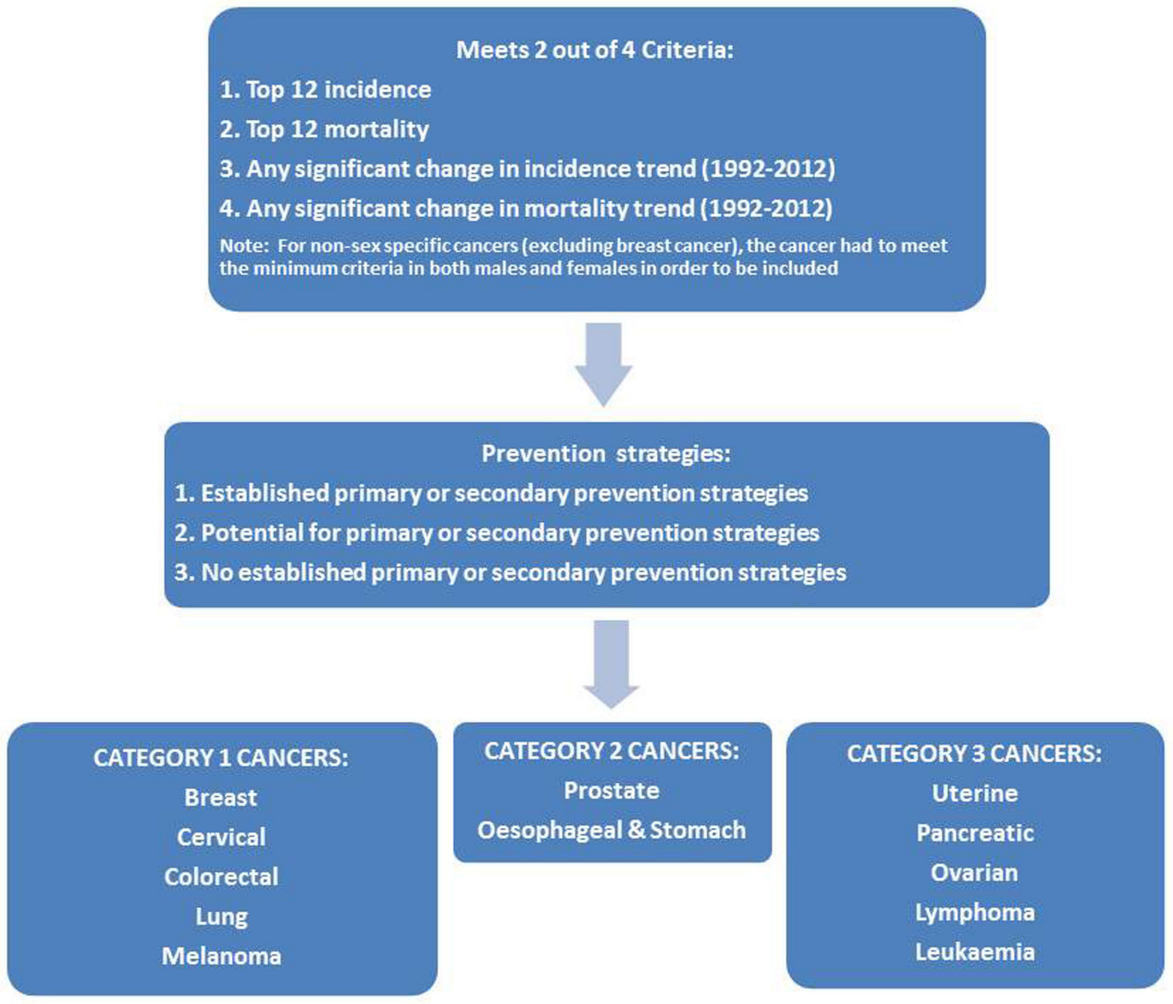
Flowchart of the process for prioritizing cancers with both the biggest impact and the best opportunities for prevention in Western Australia.

Using incidence and mortality data, the list of 55 cancers was refined based on meeting at least two of four possible criteria (Figure 1):

- Top 12 for incidence rate (2012).
- Top 12 for mortality rate (2012).
- Statistically significant change in incidence trend (1992–2012).
- Statistically significant change in mortality trend (1992–2012).

For non-sex-specific cancers (excluding breast cancer), the cancer had to meet the minimum criteria in both males and females to be considered for inclusion.

Applying these criteria reduced the list of 55 cancers to 12 cancers considered to have a large impact on the WA community: breast, cervical, ovarian, uterine, prostate, colorectal, leukemia, lung, lymphoma, melanoma, pancreatic, and esophageal/stomach cancer. The unknown primary site category also met the criteria, but this category was excluded from the list due to a lack of specificity regarding these cancers (21).

## Considering Opportunities for Prevention

The project then considered the cancers in terms of their potential preventability. However, operationalizing the concept of preventability also proved challenging. What was meant by a “preventable” cancer? Did this mean completely preventable, mostly preventable or just partially preventable? Should it be preventable in the whole population or only in specific high-risk groups? How would population-based screening and early detection as forms of secondary prevention fit into any “preventability” definition? And importantly, at what level of “preventability” should public health action be triggered?

The scientific literature was reviewed for paradigms of preventability. Although a body of literature is available, only one paper was located that attempted to operationalize the concept (8, 22–24). Smith et al. (8) defined three categories of preventability: “*all or mostly preventable*” if 50% or more of cases were considered preventable, “*sometimes preventable*” for 20–49% and “*rarely preventable*” for less than 20%, although it is not clear what data were used to estimate the proportion of preventability. No papers were identified that provided a recommendation for the level of preventability that should trigger public health action. The resulting discussion among the expert advisory group and project staff led to the development of three categories:

1. Clearly established strategies for primary or secondary prevention (defined as known modifiable risk factors accounting for more than half of cases and/or a viable and recommended population-based screening program exists).
2. No clearly established strategies for primary or secondary prevention, but potential for targeted primary or secondary prevention in population subgroups.
3. No clearly established strategies for primary or secondary prevention.

## Combining Information on Impact and Preventability

For the 12 high impact cancers, the literature was reviewed to identify: (i) strong evidence and/or scientific consensus regarding modifiable risk factors; (ii) data on population attributable fractions for the risk factors that might be relevant to the WA context; (iii) information on population-based screening programs that were currently operating or proposed for WA; and (iv) any evidence to support specific screening programs in particular populations and/or high-risk groups.

The International Agency for Research on Cancer was the source for much of the information on risk factors (25–28), while data for the population attributable fractions came primarily from one large study conducted in the UK (5). Population-based screening programs for bowel, breast, and cervical cancer are operating in WA (12–14). Evidence was found to suggest possible screening strategies for three cancers, lung (in heavy smokers), prostate, and esophageal/stomach (among certain Asian populations and/or groups with high consumption of salt-preserved foods) (29, 30).

This literature review process was used to classify the 12 cancers in terms of the three categories of preventability for the WA context. Cancers with clearly established strategies for primary or secondary prevention (category 1) included breast, cervical, colorectal, lung, and melanoma. Cancers with potential for targeted primary or secondary prevention in population sub-groups (category 2) included prostate and esophageal/stomach. Cancers with no clearly established strategies for primary or secondary prevention (category 3) included uterine, pancreatic, ovarian, and leukemia. The seven cancers that were determined to have both a high impact and also some potential for prevention (category 1 and 2 cancers) became our priority cancers for phase two of the project.

The source data behind identifying the cancers with the greatest impact and combining impact and preventability is summarized in two publically available reports called “Choosing Cancers for Your Say on Cancer in WA” and “The data behind Your Say on Cancer in WA” which are both available from: http://www.healthywa.wa.gov.au/yoursayoncancerwa.

## Further Work

The second phase of the project went on to present data on our seven priority cancers to a public consultation on preferences for cancer control. The results of this consultation and the agenda for action that arose out of it were published as the Chief Health Officer’s Report, “*Priorities and Preferences for Cancer Control in Western Australia*” (31) and are available online at: http://www.healthywa.wa.gov.au/yoursayoncancerwa.

## Discussion

The value of this paper lies in describing a method for priority setting that attempts to identify and unravel some of the factors that contribute to cancer prevention policy-making, but which are rarely made explicit. For example, this project examined a wide range of statistics that could be used to determine the relative impact of different cancers and attempted to explicitly justify why certain statistics were chosen over others. In addition, the project sought to explicitly define what might constitute a “preventable” cancer from a policy point of view to explain why some cancers were prioritized over others.

The result is a list of 12 high impact cancers, seven of which have been identified as having at least some potential for prevention (categories 1 and 2) in the Western Australian context. The collaborative nature of our project has ensured that the selection of these seven cancers as priority cancers has widespread acceptance among the cancer prevention community in WA. The second phase of this project involved community consultation around the seven potentially preventable cancers to get a sense of the public’s knowledge of, and their preferences and priorities for, public health action addressing these specific cancers/risk factors. This process found that respondents were generally surprised by the preventability of cancer overall, and in particular, the preventability of bowel cancer and cervical cancer. Red and processed meat intake, alcohol consumption, and salt were also identified as clear areas for increased community education in the immediate future (31). We have already engaged with our health promotion partners in reviewing the evidence-based messages for these areas. Work to determine the most efficient and cost-effective prevention strategies in these areas is an important next step and the process of developing, delivering, and evaluating programs will be ongoing.

The strength of this project is that the methods can be readily replicated by others who seek to identify cancer prevention priorities in different settings. In addition, the involvement of an expert advisory group at every stage was beneficial in providing context and perspective to cancer-related data, as well as building relationships across the relevant disciplines. Access to high-quality data that were specific to the WA community for a large range of cancer types was a strength of our assessment of cancer impact.

Limitations included the following: first, a lack of certain types of information, in particular up-to-date Burden of Disease information and costings for some of the less common cancers. Second, the arbitrary cutoff of including only the top 12 for incidence and mortality rate, however the ranking of the top 25 cancers (mortality and incidence, male and female separately) was documented for transparency and is available from http://www.healthywa.wa.gov.au/yoursayoncancerwa. Third, the absence of any clear definition of preventability in cancer prevention. The use of a 50% cutoff for known modifiable risk factors in defining a preventable cancer was based on limited evidence, and the heavy reliance on UK data sources for the population attributable fractions was suboptimal. The subsequent publication of a study estimating Australian population attributable fractions supports some, but not all, of the UK study’s findings. Most notably, the Australian study estimated that only 63% of Melanomas were due to exposure to UV radiation, as opposed to 86% of melanomas in the UK (4, 5). However, even the lower population attributable fraction of 63% would not have changed the classification of melanoma in this study as a category 1 “preventable” cancer. Differences also occurred in population attributable fractions for ovarian cancer and leukemia, but these were of a smaller magnitude, and again would not have changed the classification of the cancers in this study (4, 5).

An important gap in this research topic more broadly is the lack of discussion around what level of “preventability” should trigger public health action for cancer. This is in contrast to other areas within public health, for example, infectious diseases management, where there are published guidelines for the level of public health threat which stimulates action (32, 33).

## Conclusion

Cancer will continue to be a leading cause of ill health and death, unless we can capitalize on the potential for 30–40% of these cancers to be prevented. In this light, cancer prevention represents an enormous opportunity for public health. However, there is a challenge for governments in prioritizing cancer prevention targets, especially in financially constrained environments. This paper describes a process for synthesizing information from detailed cancer statistics, expert opinion, and the published literature to help identify cancer prevention priorities. The strength of our approach is that it is transparent, reproducible, and applicable to other settings. The result is a list of 12 high impact cancers in adults, 7 of which have been identified as having at least some potential for prevention in the Western Australian context. This list has been used to justify and drive further work around cancer prevention opportunities and priorities in WA. Broader discussion around defining preventability and initiating prevention actions for cancer would contribute to international efforts to reduce the incidence and impact of these diseases globally.

## Ethics Statement

Approval for the project and access to the de-identified data were granted by the Western Australian Cancer Registry Data Custodian, in accordance with our institutional ethics committee terms of reference.

## Author Contributions

JG and LM assisted with study design, conducted the study including data extraction and analysis, reviewed the literature, and wrote the manuscript. TW conceptualized the project, chaired the expert advisory group, and reviewed the manuscript. TA, MD, PK, DR, TS and TT constituted the expert membership of the advisory group and provided advice and supervision on study design and analysis, data interpretation, and reviewed the manuscript.

## Conflict of Interest Statement

The authors declare that the project was conducted in the absence of any commercial or financial relationships that could be construed as a potential conflict of interest.
